# Evaluation of the semen microbiome for fertility in men with obesity using next-generation sequencing

**DOI:** 10.1186/s12610-025-00294-x

**Published:** 2025-12-05

**Authors:** Elzem Nisa Alkan, Neslihan Hekim, Sezgin Gunes, Ramazan Asci, Ralf Henkel

**Affiliations:** 1 Department of Medical Biology, Faculty of Medicine, Ondokuz Mayis University, Samsun, 55139 Turkey; 2 Stem Cell Application and Research Center, Ondokuz Mayis University, Samsun, Turkey; 3 Department of Urology, Faculty of Medicine, Ondokuz Mayis University, Samsun, Turkey; 4 LogixX Pharma, Reading, Berkshire England, UK; 5 Department of Medical Bioscience, University of the Western Cape, Bellville, South Africa

**Keywords:** Male infertility, Microbiome, Obesity, Sperm DNA fragmentation, Total antioxidant capacity, Sperm chromatin condensation, Infertilité masculine, Microbiome, Obésité, Fragmentation de l’ADN des Spermatozoïdes, Capacité antioxydante totale, Condensation de Chromatine des Spermatozoïdes

## Abstract

**Background:**

The study aimed to evaluate the microbial content and diversity in semen samples of men with obesity, determine the differences between infertile and fertile groups, and investigate the effect of seminal microbiota on semen parameters, sperm DNA fragmentation, sperm chromatin condensation, and total antioxidant capacity.

**Results:**

The study included thirteen infertile men with obesity as subjects and five fertile men with obesity as the control group (aged 18–55 years, body mass index > 30 kg/m²). The most abundant bacteria in both groups were seen to be belonging to the phylum of *Bacillota*,* Pseudomonadota*,* Actinomycetota* and *Bacteroidota*. The most common bacteria at the genus level were *Pseudescherichia*,* Staphylococcus*,* Paenibacillus*,* Streptococcus*,* Klebsiella*, and *Moraxella*, which had similar distributions in both groups. A negative correlation was observed between the percentage of aniline-positive sperm and motility (*p* < 0.0001), sperm concentration (*p* = 0.0001) and total sperm count (*p* = 0.001). It was found that *Brevibacterium*,* Paenibacillus*,* Alistipes*,* Lactiplantibacillus*,* Rhizobacter*,* Sphingomonas* and *Venlonella* genera were correlated with sperm DNA fragmentation; *Pantoea*,* Devosia*,* Bacteroides*,* Acidovorax* were correlated with total antioxidant capacity, *Fusobacterium* was correlated with the histone-rich sperm, and *Corynebacterium*,* Hydrogenophaga*, and *Paenalcagenes* were associated with body mass index.

**Conclusion:**

Bacterial species in semen may play a role in male infertility by affecting semen quality, sperm DNA fragmentation or total antioxidant capacity.

**Supplementary Information:**

The online version contains supplementary material available at 10.1186/s12610-025-00294-x.

## Résumé

**Contexte:**

L’étude visait à évaluer le contenu microbien et la diversité des échantillons de sperme d’hommes obèses, à déterminer les différences entre les groupes infertiles et fertiles, et à étudier l’effet du microbiote séminal sur les paramètres du sperme, la fragmentation de l’ADN des spermatozoïdes, la condensation de la chromatine des spermatozoïdes, et la capacité antioxydante totale.

**Résultats:**

L’étude a inclus treize hommes infertiles obèses comme sujets et cinq hommes fertiles obèses comme groupe témoin (âgés de 18 à 55 ans, indice de masse corporelle >30 kg/m²). Les bactéries les plus abondantes dans les deux groupes appartenaient à l’embranchement des *Bacillota*, *Pseudomonadota*, *Actinomycetota* et *Bacteroidota*. Les bactéries les plus courantes au niveau du genre étaient *Pseudescherichia*, *Staphylococcus*, *Paenibacillus*,* Streptococcus*, *Klebsiella *et *Moraxella*, qui avaient des distributions similaires dans les deux groupes. Une corrélation négative a été observée entre le pourcentage de spermatozoïdes positifs à l’aniline et la motilité (*p *< 0,0001), la concentration de spermatozoïdes (*p* = 0,0001) et le nombre total de spermatozoïdes (*p* = 0,001). Il a été constaté que les genres *Brevibacterium*, *Paenibacillus*, *Alistipes*, *Lactiplantibacillus*, *Rhizobacter*, *Sphingomonas* et *Venlonella* étaient corrélés avec la fragmentation de l’ADN des spermatozoïdes ; *Pantoea*, *Devosia*, *Bacteroides*, *Acidovorax* étaient corrélés avec la capacité antioxydante totale, *Fusobacterium* était corrélé avec les spermatozoïdes riches en histones, et *Corynebacterium*, *Hydrogenophaga* et *Paenalcagenes* étaient associés à l’indice de masse corporelle.

**Conclusion:**

Les espèces bactériennes présentes dans le sperme peuvent jouer un rôle dans l’infertilité masculine en affectant la qualité du sperme, la fragmentation de l’ADN des spermatozoïdes ou la capacité antioxydante totale.

## Introduction

Male infertility is a highly heterogeneous disorder affected by both genetic and environmental factors. Studies show a significant decrease in sperm parameters over the years [1]. It is considered that changes in lifestyle, nutrition and environmental conditions play an important role in the increasing incidence of male infertility [2]. Another contributing factor to infertility that is significantly affected by nutrition is the microbiome. It has been shown that the microbiome plays an important role, primarily in the immune system and metabolic processes, and may be associated with many health conditions, especially inflammatory diseases such as obesity [3, 4]. Research reveals that obesity is linked to a specific microbiota profile and their composition may predispose to obesity [5, 6].

Obesity is known to be closely linked to infertility as it causes hormonal disorders, disruptions in spermatogenesis, increased testicular temperature, low sperm quality, sperm DNA fragmentation (SDF) and erectile dysfunction [2, 7, 8].

Studies investigating the relationship between male infertility and microbiota are quite limited [9–11]. An important reason for this is that only until recently there was the belief that the urogenital tract is sterile [12, 13]. With advancements in sequencing technologies, it has been observed that the urogenital tract and semen harbor a wide diversity of microbes, even in healthy men. While some of the studies comparing the seminal microbiomes of fertile and infertile men could not detect a significant difference between these two groups, some studies show that the microbial content and diversity in semen may be associated with sperm parameters and, therefore, infertility [14–16]. Although various studies reveal the effects of obesity on the microbiota, they have generally been centred the intestinal microbiota [17]. When examining the triadic relationship between male infertility, microbiota, and obesity, far fewer studies are available. Ding et al. (2019) found that transferring fecal microbes from high-fat diet-fed mice to mice on a regular diet significantly reduced sperm count and motility [18]. Similarly, a recent study on zebrafish demonstrated that obesity not only severely decreased sperm motility but also disrupted testicular microbiota composition [19]. Disruptions in the urogenital microbiota, associated with inflammation, may negatively impact sperm parameters. Considering the evidence on the obesity-infertility relationship, it is plausible to hypothesize that obesity may affect the seminal microbiota and thus have an impact on fertility.

This study aimed to investigate the potential role of seminal microbiota, with a focus on obesity-related alterations, in male infertility. In this regard, the seminal microbiomes of fertile and infertile individuals with obesity were compared, and the effects of the seminal bacteria on semen parameters, SDF, chromatin condensation and total antioxidant capacity were evaluated.

## Materials and methods

### Subjects

The study groups consist of infertile men attended the Urology Clinic of Medical Faculty, Application and Research Hospital, for in vitro fertilization treatment. The patient group consisted of thirteen volunteers aged 18 to 55 years with a body mass index (BMI) above 30 kg/m^2^. The control group included five men with children under two years of age and a BMI of 30 kg/m^2^ or more. Their ages were matched to the paient group within a ± 5 year age.

In the patient group, care was taken to ensure that infertility was caused solely due to male factors. All female partners were healthy, had a normal obstetric examination, and were between 18 and 55 years of age. Infetile men with known genetic causes such as chromosome anomaly, Y-chromosome microdeletion and cystic fibrosis were excluded from the study.

The study was approved by the Ethics Committee of Ondokuz Mayis University, Faculty of Medicine (OMU KAEK: 2022/139) and informed consent was obtained from all participants.

### Semen analysis

Semen samples used for this study were obtained by masturbation following 3–5 days of sexual abstinence. After the samples were liquefied by incubating them at 37 °C for 20–30 min, semen analysis was manually performed according to World Health Organization guidelines [20] at the Andrology laboratories of the OMU Faculty of Medicine Application and Research Hospital. Sperm morphology and leukocyte count were evaluated on fixed smears using the Papanicolaou staining method in accordance with the overall procedure recommended in the manual. Each semen samples was divided into two aliquots. One aliquot was used to evaluate SDF via the TUNEL assay, to measure TAC, and to assess sperm chromatin condensation quality by using aniline blue staining. The second aliquot was reserved for bacterial DNA isolation from seminal plasma.

### Preparation of samples

All aliquots were centrifuged at 500xg for 7 min to precipitate the sperm cells. The supernatant, consisting of seminal plasma, was divided into 500 µl aliquots and stored at −80 °C until further analysis.

The semen pellet intended for TUNEL analysis was washed once with 1x phosphate-buffered saline (PBS) (Gibco, NY, USA). The washing process was performed by adding 1 ml of 1xPBS to the pellet, gentle pipetting, and then centrifuging at 500xg for 7 min. Following centrifugation, the supernatant was discarded.

The pellet was then resuspended in 100 µl 1x PBS, followed by addition of 900 µL of 3.6% paraformaldehyde (PFA) (Merck KGaA, Darmstadt, Germany), stored at + 4 °C for a minimum of 2 days. PFA was removed within 10 days of fixation. To remove PFA, the samples were centrifuged at 1,500xg for 10 min.

After discarding the supernatant, the pellet was washed by resuspending in 1 mL 1x PBS, fallowed by centrifugation at 1,500xg for 10 min and the supernatant was removed again. Finally, the pellet was homogenized in 1 mL of 1xPBS and stored at −20 °C until analysis.

The semen pellet designated for aniline blue staining was washed twice with 1x PBS, using the same procedure as applied for the TUNEL assay. Following the removal of the final supernatant, the pellet was resuspended in 10–15 µL of 1xPBS. Two slides were prepared and labelled for each sample. A small volume of the resuspended samples were dropped onto each slide and smears were prepared using a coverslip. Once completely air-dried, the slides were fixed in 3% glutaraldehyde solution.

### Assessment of aniline blue staining

Sperm chromatin condensation was examined by employing the aniline blue staining method [21]. Fixed slides were stored at +4 °C and stained within a few days. For stainng, slides were immersed in a 5% aniline blue (Sigma, Steinheim, Germany) solution for 15 min. Excess dye was then removed from the slides under tap water. After drying, the slides were examined under a light microscope at 100x magnification (CX31, Olympus Life and Material Sciences, Hamburg, Germany). Spermatozoa that stained dark blue were classified as aniline-positive, indicating abnormal chromatin condensation, while pale blue or unstained were considered aniline-negative, indicating normal chromatin condensation. A minimum of 200 spermatozoa were evaluated per slide [22].

### TUNEL analysis

Sperm DNA fragmentation was measured using the TUNEL assay with the In Situ Cell Death Detection kit (Roche Diagnostics, Mannheim, Germany) [23].

#### Preparation of sample material

Following preliminary processing, sperm were smeared on poly-L-lysine (Sigma, Missouri, USA) coated slides and incubated for one hour at 60 °C to enhance adhession. Poly-L-lysine coated slides were divided into two regions and a few drops of phosphate-buffer sucrose solution (Bio Basic, Markham, Canada) were added to each region to promote adherence of the sample. Aliquots of 300 µL were applied to the slides and incubated overnight at + 4 °C in a humidified chamber [24].

#### Permeabilization of sample material

Subsequently, the slides were washed twice with PBS and then examined under a light microscope. A fresh prepared permeabilization solution containing 0.1% sodium citrate (Surechem, Suffolk, England) and 0.1% Triton X-100 (VWR Life Science Amresco, Solon, USA) was used for the permeabilization process. After the solution was applied to the sample, the slides incubated at + 4 °C for 10 min. After permeabilization, the slides were washed once and left to air dry [24].

#### Labeling and TUNEL reaction, screening and image analysis

The TUNEL reaction mixture was prepared immediately before use and all procedures were carried out in the dark. A total of 100 µl of labeling solution was set aside for the negative controls. The remaining labeling solution was combined with entire enzyme solution to prepare TUNEL reaction mixture. Each included a negative control without TdT enzyme and a positive control using recombinant DNase I. For the labeling step, slides were first washed twice with PBS. Thereafter, 50 µl of TUNEL reaction mixture were added to the selected area and slides incubated in a dark and humid chamber at 37 °C for 1 h. Following the incubation, the slides were washed three times with 1xPBS. An antifade mounting medium containing 4’,6-diamidino-2-phenylindole dihydrochloride (DAPI) (Fluoroshield, Sigma, Missouri, USA) was applied each sample and the samples covered with a coverslip [25].

The prepared samples were examined under a fluorescence microscope at 40X magnification using (BX51, Olympus Life and Material Sciences, Hamburg, Germany). Images of both DAPI and fluorescein isothiocyanate (FITC) signals were captured, corresponding to excitation wavelengths of 461 nm and 519 nm, respectively. Sperm showing FITC signal were considered DNA-fragmented. For each sample, images of at least five separate areas were taken and an average of 500 cells was counted. DAPI and FITC signals in each area were counted separately using the Image J program on a computer. The DNA Fragmentation Index (DFI) of viable sperm was calculated using the the formula (FITC/DAPI) x 100 [24].

### Assement of total antioxidant capacity

TAC of the seminal plasma was measured ELISA method with commercial the antioxidant assay kit (Cayman Chemical, Michigan, USA) [26]. All reagents provided in the kit were prepared according to the manufacturer’s instructions. Samples that were stored at −80 °C were thawed at room temperature and subsequently centrifuged at 10,000xg for 8 min. All seminal plasma samples were diluted at 1:20. Trolox (standart), chromogen and metmyoglobin solutions were prepared using ultrapure water (Wisent, Québec, Canada). All reagents and diluted samples were added to microplate wells in accordance with the assay protocol. The reaction was initiated by adding 200 mM H_2_O_2_ to each well, resulting in the formation of a blue/green product. The color intensity, corresponding to antioxidant capacity, was measured spectrophotometrically at 405 nm and 750 nm using a Multiskan GO microplate reader (Multiskan GO, ThermoFisher Scientific, Vantaa, Finland). The antioxidant concentration of the sample was calculated by inserting the mean absorbance values into the equation derived from linear regression of Trolox standard curve. Each sample from the patient group were analysed in duplicate, while control group samples were studied in triplicate. The total antioxidant concentrations of each sample were calculated using the following formula:

$$\begin{aligned} &Antioxidant\;(mM)\\&=\left[\frac{\left(Average\;absorbance\;of\;the\;sample\right)-\left(y-intercept\right)}{Slope}\right]x\;Dilution \end{aligned}$$

### Microbiome analysis

Microbiome profiling was conducted using next-generation sequencing (NGS). Semen samples aliquots designed for microbiome analysis were stored at −80 °C until use. All samples were transported to the analysis laboratory under cold chain conditions. After bacterial DNA isolation from the samples, the 16 S rRNA gene was amplified by PCR using region-specific primers targeting the V3–V4 hypervariable regions [27]. Subsequently, library preparation was performed, and high-throughput sequencing was conducted on the NovaSeq 6000 platform employing the sequence-by-synthesis method (Illumina, California, USA). Bioinformatic analysis was carried out using the QIIME2 platform (Knight Lab, California, USA) for taxonomic classifications.

### Statistical analysis

Statistical analysis of the data was performed with the MedCalc Statistical Software version 19.2.6 (MedCalc Software bv, Ostend, Belgium). The Shapiro-Wilk test was employed to evaluate normality of data distribution.

For the data with normally distribution, comparison between groups was made using the independent samples t-test. For non-normally distributed data, the Mann-Whitney U test was applied. Correlations between variables were assesed using Spearman Rank’s correlation coefficient. A p-value of less than 0.05 was considered statistically significant.

## Results

No difference were found between the patient and control groups in terms of age, weight, height and BMI (Supplementary Table 1).

### Semen parameters

Semen analysis data of the participants are presented in additional file 4 [see Additional file 4]. Significant differences were detected between the patient and control groups for sperm concentration (*p* = 0.043), total sperm count (*p* = 0.032), total progressively motile sperm count (*p* = 0.028), percentage of immotile sperm (*p* = 0.012), and normal sperm morphology (*p* = 0.019). No significant difference was observed for the other semen parameters (Supplementary Table 2).

### Functional analyses

**Fig. 1:**
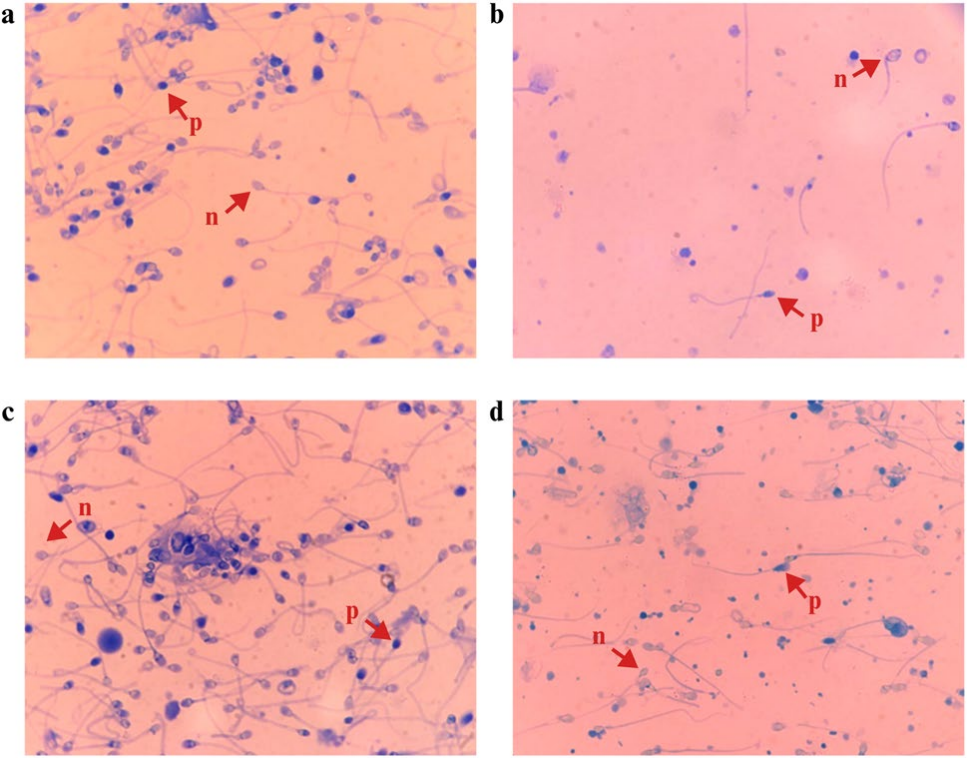
Examples of light microscope images of different participants (**a**, **b**, **c**, **d**) after aniline blue staining. Those marked with a letter p represent aniline-positive sperm cells stained with aniline dye, while those marked with an n represent unstained aniline-negative cells. Because aniline is a dye that binds to histone proteins, p cells represent histone-rich sperm, and n cells represent protamine-rich sperm

**Fig. 2:**
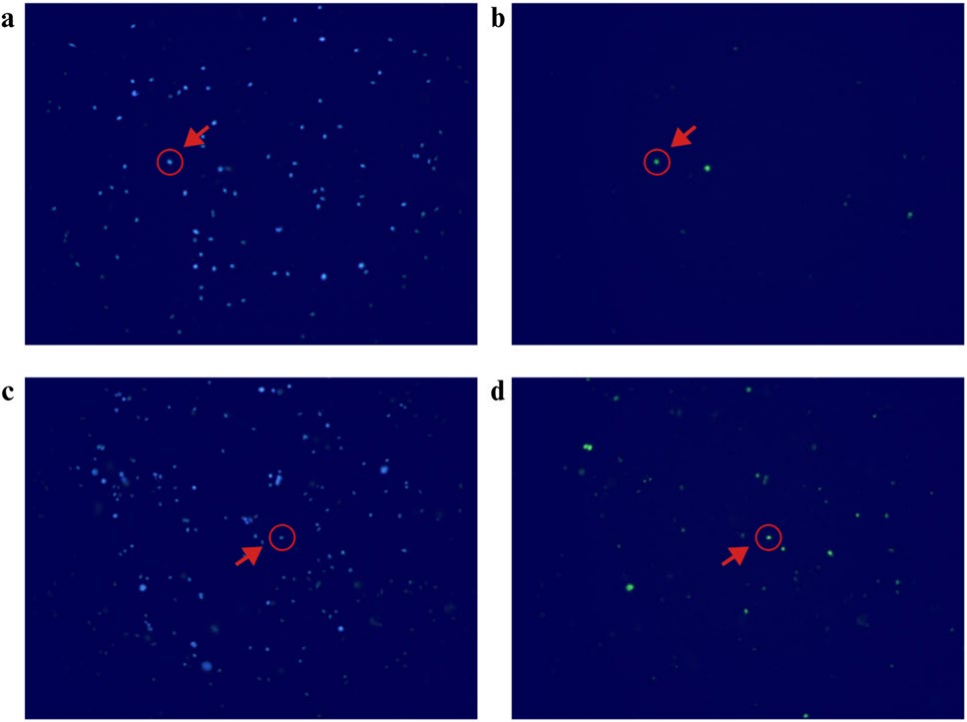
Fluorescent microscope image of DAPI and FITC signals of two different samples. The images on the left (**a**, **c**) show signals from sperm cells stained with DAPI, a nuclear dye. The right panel (**b**, **d**) displays FITC signals resulting from the binding of fluorescently labeled dUTP to the free 3’-OH ends in fragmented DNA in the same area. In two different samples, DAPI and FITC signals corresponding to a single sperm cell with DNA fragmentation are indicated by arrows

Assessment of sperm chromatin condensation using aniline blue staing was performed in 14 participants (9 patients and 5 controls) (Fig. 1). The TUNEL assay was performed on samples from 14 of the 18 participants, and SDF indexes were calculated (Fig. 2). TAC was dertermined in the seminal plasma of 17 of the 18 participants (12 patients, 5 controls). No statistically significant difference was detected between the groups (Supplementary Table 3) Detailed analysis results are provided in additional file 5 [see Additional file 5].

### Correlations between semen parameters

Table 1 presents the correlations among semen parameters. As expected, strong positive correlations were found between motility and normal sperm morphology, as well as sperm concentration and total sperm count. Normal sperm morphology was also showed a strong positive assosiation with sperm concentration, while its association with BMI, though significant, was notably weaker and negative.

**Table 1:** Correlation table showing the relationships between age, BMI and semen parameters

		Age	BMI	Motility	Morphology	Sperm Concentration	Total Sperm Count
Age	*ρ* factor	1	0,187	0,032	0,079	0,089	0,091
	*p* value		0,457	0,911	0,780	0,751	0,747
BMI (kg/m^2^)	*ρ* factor	0,187	1	−0,362	−0,518	−0,437	−0,403
	*p* value	0,457		0,185	0,048*	0,103	0,137
Motility	*ρ* factor	0,032	−0,362	1	0,897	0,942	0,889
	*p* value	0,911	0,185		< 0,0001***	< 0,0001***	< 0,0001***
Morphology	*ρ* factor	0,079	−0,518	0,897	1	0,936	0,895
	*p* value	0,780	0,048*	< 0,0001***		< 0,0001***	< 0,0001***
Sperm Concentration	*ρ* factor	0,089	−0,437	0,942	0,936	1	0,970
	*p* value	0,751	0,103	< 0,0001***	< 0,0001***		< 0,0001***
Total Sperm Count	*ρ* factor	0,091	−0,403	0,889	0,279	0,970	1
	*p* value	0,747	0,137	< 0,0001***	0,314	< 0,0001***	

Age, body mass index (BMI), and semen parameters such as motility, morphology, sperm concentration, and total sperm count were correlated in both groups. Positive correlations were observed among motility, normal morphology, sperm concentration, and total sperm count, while morphology showed a weaker negative association with BMI

*Spearman Correlation Analysis (*α* = 0.05) (Statistically significant expressions are indicated with * for < 0.05, and *** for < 0.001.)

### Microbiome analysis

Microbiome analysis was conducted on samples from 16 of the 18 study participants. Two samples could not be analyzed for microbiome due to insufficient microbial DNA concentration. Bacterial composition of semen samples were examined at the phylum [see Additional file 6], class [see Additional file 7], order [see Additional file 8], family [see Additional file 9] and genus levels [see Additional file 10]; findings are presented at the phylum and genus levels. Microbiome analysis was evaluated based on the relative abundances of bacteria. The relative abundances were determined by normalizing the abundance of each bacterial taxon to the total bacterial abundance, and these values were subsequently ranked.

#### Phylum level

**Fig. 3:**
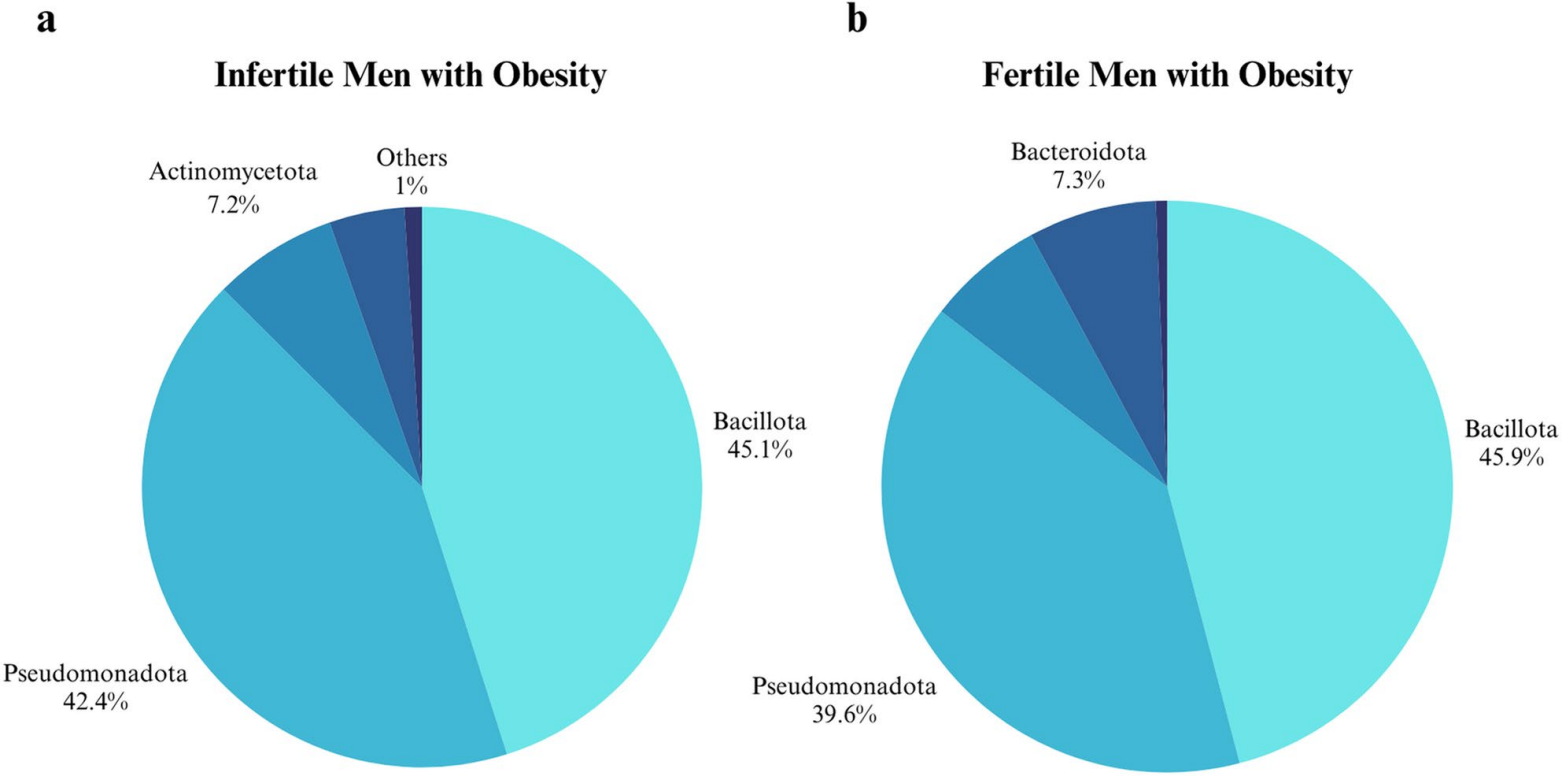
Relative abundance distributions of bacterial phyla in the semen of patient (**a**) and control (**b**) groups. The most common bacterial phyla in both groups are shown. Phyla with less than 1% are not included. The four most prevelant bacterial phyla, *Bacillota*, *Pseudomonadota*, *Actinomycetoda*, and *Bacteriodota* were detected in both infertile and fertile men with obesity. *Bacillota* and *Pseudomonadota* exhibited similar distribution percentages between the groups. *Bacteroidota* in the control group and *Actinomycetota* in the patient group were more prominent than the other group

The four most prevelant bacterial phyla, *Bacillota*, *Pseudomonadota*, *Actinomycetoda*, and *Bacteriodota* were detected in both infertile and fertile men with obesity. *Bacillota* and *Pseudomonadota* exhibited similar distribution percentages between the groups (Fig. 3). *Bacteroidota* in the control group and *Actinomycetota* in the patient group were more prominent than the other group. However, these differences did not reach statistical significance at the phylum level.

#### Genus level

**Fig. 4:**
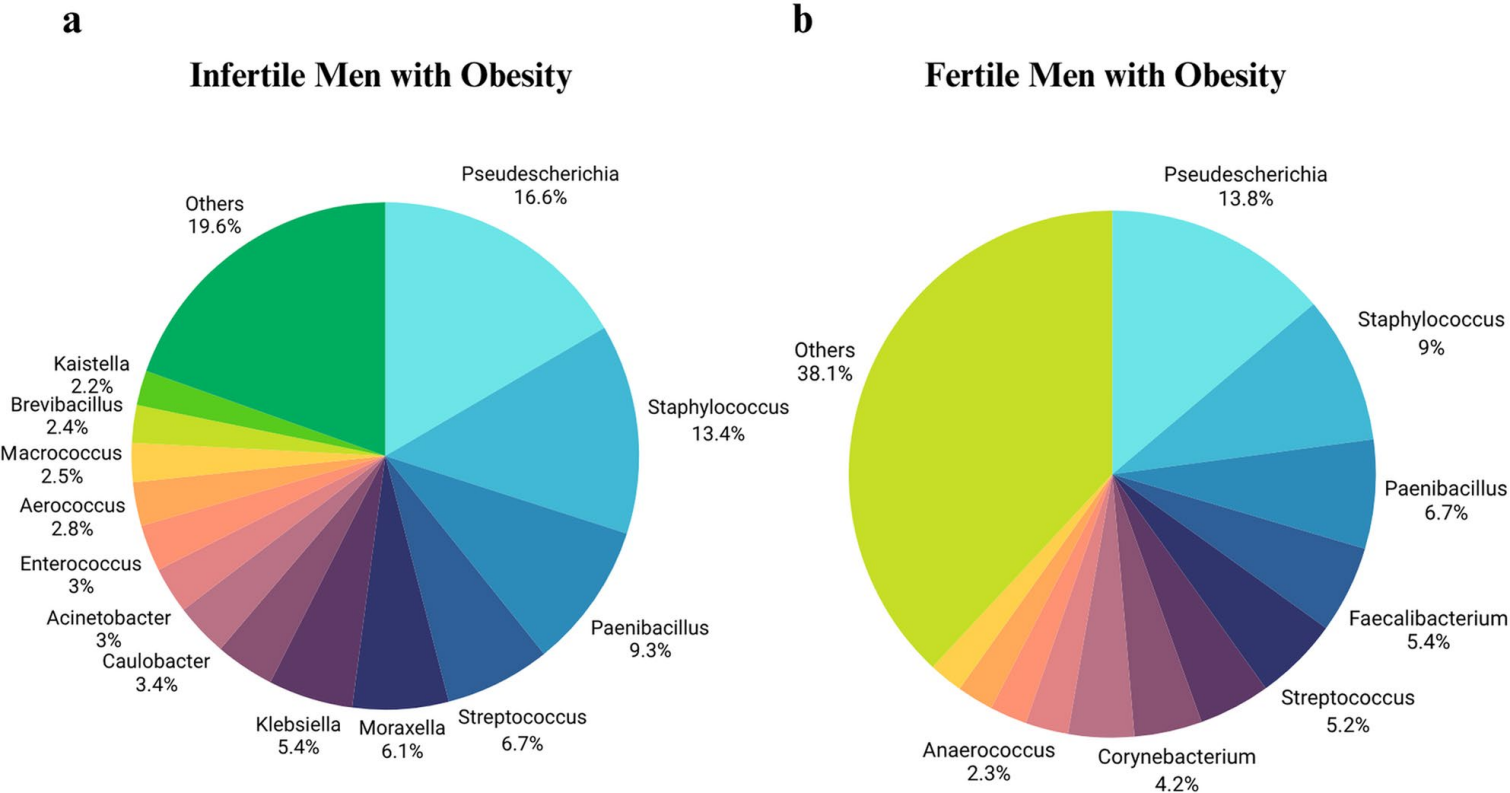
Relative abundance distributions of bacterial genera in the semen of patient (**a**) and control (**b**) groups. The most common bacterial genera in both groups are shown. A total of 206 different bacterial genera were identified across all semen sample. Genera with less than 1% are not included. The most abundant bacterial genera in both groups were *Pseudescherichia*, *Staphylococcus*, *Paenibacillus*, *Streptococcus*, *Klebsiella* and *Moraxella*. In the patient group, the relative abundance of the top genera was as follows: *Pseudescherichia*, *Staphylococcus*, *Paenibacillus*, *Streptococcus*, *Moraxella*, and *Klebsiella*. In the control group, predominant genera included *Pseudescherichia*, *Staphylococcus*, *Paenibacillus*, *Faecalibacterium*, *Streptococcus*, *Moraxella*, *Corynebacterium*, and *Klebsiella*

A total of 206 different bacterial genera were identified across all semen sample. The most abundant bacterial genera in both groups were *Pseudescherichia*, *Staphylococcus*, *Paenibacillus*, *Streptococcus*, *Klebsiella* and *Moraxella*. In the patient group, the relative abundance of the top genera was as follows: *Pseudescherichia* (16.6%), *Staphylococcus* (13.4%), *Paenibacillus* (9.3%), *Streptococcus* (6.7%), *Moraxella* (6.1%), and *Klebsiella* (5.4%). In the control group, predominant genera included *Pseudescherichia* (13.8%), *Staphylococcus* (9%), *Paenibacillus* (6.7%), *Faecalibacterium* (5.4%), *Streptococcus* (5.2%), *Moraxella* (4.4%), *Corynebacterium* (4.2%) and *Klebsiella* (4%) (Fig. 4).

Among the examined genera, *Acinetobacter* (*p* = 0.0009), *Aerococcus* (*p* = 0.0380), *Delftia* (*p* = 0.0091), *Kaistella* (*p* = 0.0032), *Psychrobacillus* (*p* = 0.0140), *Staphylococcus* (*p* = 0.0275) and *Stenotrophomonas* (*p* = 0.0109) were significantly more abundant in the patient group, whereas *Clostridium* (*p* = 0.0242), *Gemmobacter* (*p* = 0.012) and *Tahibacter* (*p* = 0.0381) were significantly more abundant in the control group (Table 2).

**Table 2:** Bacterial genera that differ between groups

Genus	Patients			Controls			
	*n*	Median	Average rank	*n*	Median	Average rank	*p* value
*Acinetobacter*	11	3.014	10.909	5	1.767	3.200	0.0009***
*Aerococcus*	11	2.941	10.182	5	1.755	4.800	0.038*
*Clostridium*	7	0.140	4.286	4	0.320	9.000	0.024*
*Delftia*	9	0.537	8.000	3	0.341	2.000	0.009**
*Gemmobacter*	9	0.239	5.444	5	0.683	11.200	0.012*
*Kaistella*	11	2.288	10.727	5	1.167	3.600	0.003**
*Psychrobacillus*	10	0.648	8.400	3	0.327	2.333	0.014*
*Staphylococcus*	11	13.705	10.273	5	9.627	4.600	0.027*
*Stenotrophomonas*	8	0.981	9.125	5	0.541	3.600	0.011*
*Tahibacter*	6	0.069	3.833	4	0.292	8.000	0.038*

Bacterial genera that showed statistically significant differences between the two groups are shown. Among the examined genera, *Acinetobacter*,* Aerococcus*,* Delftia*,* Kaistella*,* Psychrobacillus*,* Staphylococcus* and *Stenotrophomonas* in the patient group, whereas *Clostridium*, *Gemmobacter*, and *Tahibacter* were significantly more abundant in the control group

*Mann-Whitney U test (*α* = 0.05) (Statistically significant expressions are indicated with * for < 0.05, ** for < 0.01, and *** for < 0.001.)

### Relationships between the microbiome and other parameters

Significant negative associations were observed between *Corynebacterium*, *Hydrogenophaga*, *Paenalcaligenes*, and BMI, whereas *Fructilactobacillus* exhibited a significant positive association with age (Table 3).

**Table 3:** Correlation table showing the relationships between bacterial genera and age and BMI

Genus		BMI (kg/m^2^)	Age
*Corynebacterium* (*n* = 13)	*ρ* factor	−0.604	−0.245
	*p* value	0.029*	0.420
*Hydrogenophaga* (*n* = 4)	*ρ* factor	−1.000	0.211
	*p* value	< 0.0001***	0.789
*Paenalcaligenes* (*n* = 8)	*ρ* factor	−0.738	−0.371
	*p* value	0.037*	0.365
*Fructilactobacillus* (*n* = 6)	*ρ* factor	0.543	0.899
	*p* value	0.266	0.015*

The relationship between bacterial species and body mass index (BMI) and age has been shown. Significant negative associations were observed between *Corynebacterium*,* Hydrogenophaga*,* Paenalcaligenes* and BMI, while *Fructilactobacillus* showed a significant negative correlation with age

*Spearman Correlation Analysis (*α* = 0.05) (Statistically significant expressions are indicated with * for < 0.05, and *** for < 0.001.)

While *Bacillus*,* Delftia*,* Gemmobacter*,* Komagataeibacter*, and *Massilia* were associated with the percentage of immobile sperm; *Pseudomonas* was positively associated with total and progressive motility and *Lysinibacillus* and *Pantoea* with total sperm count. Notably, *Brevundimonas* showed a strong and highly significant positive association with motility, total sperm count and sperm concentration (Table 4). No statistically significant associations were observed between bacterial genera and either sperm morphology or leukocyte count in semen.

**Table 4:** Correlation table showing the relationships between bacterial genera and semen parameters

Genus		Motility	Progressive Sperm (%A)	Immotile Sperm (%C)	Total Sperm Count	Sperm Concentration
*Bacillus*	*ρ* factor	−0,029	−0,029	−0,829	−0,486	−0,486
	*p* value	0,957	0,957	0,042*	0,329	0,329
	*n*	6	6	6	6	6
*Delftia*	*ρ* factor	−0,394	−0,394	0,661	−0,345	−0,321
	*p* value	0,260	0,260	0,038*	0,328	0,365
	*n*	10	10	10	10	10
*Gemmobacter*	*ρ* factor	0,434	0,434	−0,722	0,203	0,287
	*p* value	0,158	0,158	0,008**	0,527	0,366
	*n*	12	12	12	12	12
*Komagataeibacter*	*ρ* factor	0,541	0,541	−0,793	0,036	0,143
	*p* value	0,210	0.210	0,033*	0,939	0,760
	*n*	7	7	7	7	7
*Massilia*	*ρ* factor	0,314	0,314	−0,624	0,196	0,196
	*p* value	0,346	0,346	0,040*	0,564	0,564
	*n*	11	11	11	11	11
*Pseudomonas*	*ρ* factor	0,714	0,714	−0,429	0,476	0,476
	*p* value	0,046*	0,046*	0,289	0,233	0,233
	*n*	8	8	8	8	8
*Brevundimonas*	*ρ* factor	1,000	1,000	−1,000	1,000	1,000
	*p* value	< 0,0001***	< 0,0001***	< 0,0001***	< 0,0001***	< 0,0001***
	*n*	4	4	4	4	4
*Lysinibacillus*	*ρ* factor	0,657	0,657	−0,143	0,886	0,600
	*p* value	0,156	0,156	0,787	0,019*	0,208
	*n*	6	6	6	6	6
*Pantoea*	*ρ* factor	0,667	0,667	−0,333	0,762	0,571
	*p* value	0,071	0,071	0,420	0,028*	0,139
	*n*	8	8	8	8	8

Correlations between parameters such as motility, percentage of progressive and immotile sperm, total sperm count and sperm concentration, which were found to be statistically significantly related to bacterial genera, were shown. *Bacillus*, *Delftia*, *Gemmobacter*, *Komagataeibacter*, and *Massilia* were linked to higher immotile sperm rates, while *Pseudomonas* correlated positively with motility parameters, and *Lysinibacillus*, and *Pantoea* with total sperm count. *Brevundimonas* showed a strong, significant positive association with motility, total count, and concentration

*Spearman Correlation Analysis (*α* = 0.05) (Statistically significant expressions are indicated with * for < 0.05, ** for < 0.01, and *** for < 0.001.)

When the relative abundances of bacterial genera were compared with DFI, TAC and the quality of chromatin condensation, it was found that *Brevibacterium*, *Paenibacillus*, *Alistipes*, *Lactiplantibacillus*, *Rhizobacter*, *Sphingomonas* and *Veillonella* were significantly correlated with the DFI. *Pantoea*, *Devosia*, *Bacteroides*, and *Acidovorax* were significantly associated with the TAC levels, while *Fusobacterium* showed a significant association with the sperm chromatin condensation quality (Table 5).

**Table 5:** Correlation table showing the relationships between bacterial genera and DFI, TAC and histone-rich sperm percentage

Genus		DFI	TAC	Histone-rich sperm %
*Brevibacterium*	*ρ* factor	0.670	0.161	0.115
	*p* value	0.012*	0.567	0.707
	*n*	13	15	13
*Paenibacillus*	*ρ* factor	0.593	0.257	−0.099
	*p* value	0.032*	0.355	0.748
	*n*	13	15	13
*Alistipes*	*ρ* factor	−0.762	0.000	−0.071
	*p* value	0.028*	1.0000	0.866
	*n*	8	9	8
*Lactiplantibacillus*	*ρ* factor	−0.629	−0.147	−0.238
	*p* value	0.028*	0.615	0.457
	*n*	12	14	12
*Rhizobacter*	*ρ* factor	−1.000	−0.200	−0.200
	*p* value	< 0.0001***	0.800	0.800
	*n*	4	4	4
*Sphingomonas*	*ρ* factor	−0.587	−0.112	0.154
	*p* value	0.045*	0.703	0.633
	*n*	12	14	12
*Veillonella*	*ρ* factor	−0.886	−0.200	0.143
	*p* value	0.019*	0.704	0.787
	*n*	6	6	6
*Pantoea*	*ρ* factor	−0.500	−0.881	−0.214
	*p* value	0.253	< 0.0001***	0.644
	*n*	7	8	7
*Devosia*	*ρ* factor	1.000	1.000	1.000
	*p* value		< 0.0001***	
	*n*	3	4	3
*Bacteroides*	*ρ* factor	0.167	0.661	0.617
	*p* value	0.668	0.038*	0.077
	*n*	9	10	9
*Acidovorax*	*ρ* factor	−0.400	1.000	0.200
	*p* value	0.600	< 0.0001***	0.800
	*n*	4	4	4
*Fusobacterium*	*ρ* factor	−0.600	−0.536	−0.943
	*p* value	0.208	0.215	0.005**
	*n*	6	7	6

Correlations between bacterial genera and DNA fragmentation index (DFI), total antioxidant capacity (TAC), and histone-rich sperm percentage has been shown. *Brevibacterium*, *Paenibacillus*, *Alistipes*, *Lactiplantibacillus*, *Rhizobacter*, *Sphingomonas*, and *Veillonella* were linked DFI; *Pantoea*, *Devosia*, *Bacteroides*, and *Acidovorax* correlated with TAC; and *Fusobacterium* was associated with chromatin condensation quality

*Spearman Correlation Analysis (*α* = 0.05) (Statistically significant expressions are indicated with * for < 0.05, ** for < 0.01, and *** for < 0.001.)

Taken together, these findings indicate that *Bacillus*, *Gemmobacter*, *Komagataeibacter*, *Massilia*, *Pseudomonas*, *Lysinibacillus*, *Alistipes*, *Lactiplantibacillus*, *Rhizobacter*, *Sphingomonas*, *Veillonella*, *Devosia*, *Bacteroides*, *Acidovorax*, and Fusobacterium exerted positive influences on sperm quality, whereas Delftia, *Brevibacterium*, and *Paenibacillus* showed negative influences (Tables 4 and 5). The effects of *Pantoea* and *Brevundimonas* on sperm quality remain unclear due to their differing associations with various parameters.

## Discussion

This strictly controlled study compared the seminal microbiome of fertile and infertile men with obesity, focusing on associations between bacterial communities and sperm parameters. The most abundant phyla identified were *Bacillota*, *Pseudomonadota*, *Actinomycetota*, and *Bacteroidota*. These findings are consistent with Yao et al. (2022), who reported similar dominant phyla—formerly known as *Firmicutes*, *Proteobacteria*, *Actinobacteria*, and *Bacteroidetes* [28]. Likewise, Garcia-Segura et al. (2022) found *Bacillota* to be the most prevalent (~ 59%), followed by *Pseudomonadota* (~ 19%), *Actinomycetota* (~ 8%), and *Bacteroidota* (~ 5%) [29].

At the genus level, we identified 206 distinct bacterial genera in the semen of fertile and infertile men with obesity. The most abundant were *Pseudescherichia*, *Staphylococcus*, *Paenibacillus*, *Streptococcus*, *Klebsiella*, and *Moraxella*. Consistent with our findings, Yao et al. (2022) also reported *Streptococcus*, *Staphylococcus*, and several other genera—including *Lactobacillus*, *Gardnerella*, and *Prevotella*—as predominant in semen [28].

Bukharin et al. (2022), in a study investigating the seminal microbiota and cytokines in semen of healthy men, reported *Staphylococcus* as the most dominant bacterial genus, while the least common genera were *Corynobacterium*, *Neisseria*, *Veillonella*, and *Enterococcus* [30]. Garcia-Segura et al. (2022), identified a total of 168 genera in semen, with the most frequently observed being *Finegoldia*, *Peptoniphilus*, *Anaerococcus*, *Campylobacter*, *Streptococcus*, *Staphylococcus*, *Moraxella*, *Prevotella*, *Ezakiella*, *Corynebacterium*, and *Lactobacillus* [29].

While similar finding were shared in these studies, notable differences were also observed. This variability can be attributed to numerous factors known to influence the composition of the microbiome, including lifestyle habits, dietary style, hygiene practices, and geographic location. Considering that this study was conducted in the Black Sea Region, Turkiye, regional influences should be taken into account when interpreting the results. Additionally, circumcision status is another variable impact the seminal microbiota composition [14].

Given the heterogeneous nature of male infertility, a small and highly homogeneous cohort was selected. Indeed, microbiome analysis results indicated that several bacterial genera differed significantly between fertile and infertile men with obesity. Among these, the genera *Acinetobacter*, *Aerococcus*, *Delftia*, *Kaistella*, *Psychrobacillus*, *Staphylococcus*, and *Stenotrophomonas* were found to be more abundant in the infertile group. In contrast, *Clostridium*, *Gemmobacter*, and *Tahibacter* were more prevalent in the fertile group.

Regardless of whether there is a difference between groups, examining the potential effects of bacterial genera on sperm parameters, the genera *Pseudomonas* and *Brevundimonas* appear to be associated with sperm motility. *Brevundimonas* was also found to be associated with higher total sperm count and sperm concentration. Similarly, *Lysinibacillus* and *Pantoea* were positively correlated with the total sperm count. Additionally, the abundance of the *Corynebacterium*, *Hydrogenophaga*, and *Paenalcaligenes* genera were negatively correlated with BMI, while the abundance of *Fructilactobacillus* increased with age.

Several studies have highlighted links between seminal microbiota and male infertility. Hou et al. (2013) proposed *Anaerococcus* as a potential marker for reduced sperm quality [10]. Weng et al. (2014) reported that *Lactobacillus* and *Gardnerella* were more abundant in normal semen, while *Prevotella* dominated low-quality samples [16]. Other research noted distinct microbial patterns in infertility phenotypes like hyperviscosity and oligoasthenoteratozoospermia, suggesting microbiota shifts and immune responses may play a role [15]. Similarly, Baud et al. (2019) reported *Prevotella* abundance in samples with abnormal spermiograms and higher *Staphylococcus* levels in normospermic men [31]. They also noted that *Lactobacillus* was more abundant in samples with normal sperm morphology. A study examining rectal swabs, urine, and semen samples from men with primary idiopathic infertility and fertile controls, *Prevotella* abundance was found to be associated with BMI and inversely correlated with the sperm concentration, while it was directly related to total motile sperm count [32]. Bukharin et al. (2022) reported that *Staphylococcus*, *Corynebacterium*, and *Enterococcus* were dominant in the semen of infertile men, and these genera were linked to enhanced biofilm formation, which may impair sperm function and contribute to infertility [30].

Moreover, in men with idiopathic infertility, bacteria from the *Paenibacillaceae*, *Lachnospiraceae* families, and the genus *Ralstonia* were associated with reduced sperm concentration, while *Peptoniphilus* correlated with low progressive motility and *Sphingomonadaceae* with decreased total motility [29]. Conversely, higher levels of *Ralstonia*, *Bacillus*, and *Steroidobacter* were linked to increased semen volume, and *Flavobacterium* was associated with higher progressive motility. *Filifactor* and *Gardnerella* showed positive correlations with total motility, and elevated *Moraxella* and *Massilia* abundance appeared to support better sperm morphology [29]. *Gardnerella* was also reported by Weng et al. to be more abundant in normal semen samples compared to those from infertile men. However, another study has associated *Gardnerella vaginalis* with reduced sperm concentration and abnormal morphology [33]. These findings indicate that different species of the same genus may exert varying influences on sperm quality. In their study examining the semen, intestinal and urinary microbiota in patients with semen abnormalities, Cao et al. (2023) reported that *Lactobacillus*, *Prevotella*, *Finegoldia*, *Staphylococcus*, *Streptococcus*, and *Ureaplasma* were predominant in semen. Among these, *Staphylococcus* was suggested as a potential pathogen, as its abundance was significantly higher in asthenozoospermic and hyperviscous semen samples [34].

Furthermore, men with abnormal sperm motility exhibited an increased abundance of *Lactobacillus iners* compared to those with normal motility [35]. Similarly, higher abundances of *Pseudomonas stutzeri* and *P. fluorescens*, and lower levels of *P. putida*, were observed in men with abnormal sperm concentration. The discrepancies between these findings and ours—as well as inconsistencies among other studies—likely reflect the multifactorial nature of microbiome influences. Moreover, male infertility encompasses diverse subtypes, and variations in study populations or lack of stratification by infertility phenotype may contribute to differing results.

SDF was positively correlated with the abundance of *Brevibacterium* and *Paenibacillus*, and negatively with *Alistipes*, *Lactiplantibacillus*, *Rhizobacter*, *Sphingomonas*, and *Veillonella*. A related study also linked *Paenibacillus* to reduced protamination and increased double-strand DNA breaks [29]. In contrast to our findings, the same study reported negative correlations between SDF and the abundance of *Moraxella*, *Brevundimonas*, and *Flavobacterium*. Regarding TAC, *Pantoea* was negatively associated, whereas *Devosia*, *Bacteroides*, and *Acidovorax* showed positive correlations.

The increased abundance of *Fusobacterium* was negatively associated with the proportion of histone-rich sperm, indicative of proper chromatin condensation, and could thus be associated with improved fertilization potential. *Fusobacterium*, typically part of the oral microbiota, includes both commensal species and strains linked to systemic conditions such as sinus and liver abscesses, skin ulcers, gastrointestinal cancers, and inflammatory bowel disease [36]. In studies investigating the origins of the seminal microbiome, it has been suggested that the gut and oral microbiota may contribute to the seminal microbiome through hematogenous and/or lymphatic dissemination [14]. Thereby it can be hypothesized that the source of *Fusobacterium* found in semen is the oral microbiota. Additionally, higher levels of *Fusobacterium* were observed in human papillomavirus (HPV)-negative semen compared to HPV-positive samples, implying a possible role in modulating semen quality [37].

One of the strengths of this study is that it was conducted on a relatively homogeneous group consisting solely of individuals with obesity. Additionally, while there are a limited number of studies examining the relationship between the seminal microbiome and infertility, this study holds unique value in assessing potential associations not only with conventional sperm parameters but also with parameters such as sperm DFI, chromatin condensation, and seminal plasma TAC.

### Study limitations

The most significant limitation of this study is the relatively small sample size. The considerable difficulty of collecting semen samples compared to many other types of specimens, combined with the high cost of procedures such as microbiome analysis, has unfortunately had a substantial negative impact on the number of participants that could be included in the study. In particular, reaching the target number of participants for the control group of fertile men with obesity, as determined by power analysis, proved to be extremely challenging. The primary reason for this difficulty is that fertile men, having already fathered children, typically do not visit urology clinics or feel the need to undergo semen analysis. The underrepresentation of fertile and infertile groups without obesity, due to similar reasons, can also be considered a limitation. Including these groups would be highly valuable for directly assessing the impact of obesity on the investigated parameters.

Another limitation is that the microbiome analysis was conducted using the 16 S amplicon sequencing method. Although widely used due to its cost-effectiveness and broad taxonomic coverage, this method lacks species-level resolution. More advanced and precise investigations may be achived through shotgun metagenome sequencing method, which allows species and even strain level resolution, as well as functional profiling [38].

## Conclusion

Although no significant differences were observed at the phylum level between the seminal microbiota of fertile and infertile men with obesity, some bacterial genera were found to differ significantly in numerous semen properties. Among these, various bacterial genera were found to be associated with SDF, TAC, and sperm chromatin condensation. When these results are evaluated together, it can be suggested that bacterial genera present in semen play a role in male infertility by affecting parameters determining semen quality such as motility, SDF or TAC. Considering the small sample size of this study, it is expected that more meaningful results could be obtained by working with a larger sample, especially by including fertile and infertile groups without obesity in the study.

As a future perspective, it is recommended to establish standardized guidelines for seminal microbiome analysis, along with more accessible and informative technologies that capture the full microbial profile. Additionally, clinical trials involving homogeneous populations are needed to better investigate male infertility, which is a rather heterogeneous disorder.

## Supplementary Information

**Additional File 1:** Supplementary Table 1. Statistical data regarding the demographic information of the patient and control groups.

**Additional File 2:** Supplementary Table 2. Statistical data regarding semen parameters of patient and control groups.

**Additional File 3:** Supplementary Table 3. Comparison of chromatin condensation, DFI and TAC between groups.

**Additional File 4.** Semen analysis data for each participant.

**Additional File 5.** Detailed results of aniline staining, ELISA and TUNEL analyses.

**Additional File 6.** Phylum level microbiome analysis results for each participant.

**Additional File 7.** Class level microbiome analysis results for each participant.

**Additional File 8.** Order level microbiome analysis results for each participant.

**Additional File 9.** Family level microbiome analysis results for each participant.

**Additional File 10.** Genus level microbiome analysis results for each participant.

## Data Availability

The datasets used and/or analysed during the current study are available from the corresponding author on reasonable request.

## Abbreviations

BMI: Body mass index
DFI: DNA fragmentation index
ELISA: Enzyme linked immunosorbent assay
HPV: Human papillomavirus
PBS: Phosphate-buffered saline
PCR: Polymerase chain reaction
PFA: Paraformaldehyde
SDF: Sperm DNA fragmentation
TAC: Total antioxidant capacity
TUNEL: Terminal deoxynucleotidyl transferase (TdT) dUTP nick-end labeling

## References

[1] Levine H, Jorgensen N, Martino-Andrade A, Mendiola J, Weksler-Derri D, Mindlis I, et al. Temporal trends in sperm count: a systematic review and meta-regression analysis. Hum Reprod Update. 2017;23(6):646–59. 28981654 10.1093/humupd/dmx022 PMC6455044

[2] Leisegang K, Sengupta P, Agarwal A, Henkel R. Obesity and male infertility: mechanisms and management. Andrologia. 2021;53(1):e13617. 32399992 10.1111/and.13617

[3] Meldrum DR, Morris MA, Gambone JC. Obesity pandemic: causes, consequences, and solutions-but do we have the will? Fertil Steril. 2017;107(4):833–9. 28292617 10.1016/j.fertnstert.2017.02.104

[4] Young VB. The role of the microbiome in human health and disease: an introduction for clinicians. BMJ. 2017;356:j831. 28298355 10.1136/bmj.j831

[5] Leocadio PCL, Oria RB, Crespo-Lopez ME, Alvarez-Leite JI. Obesity: more than an inflammatory, an infectious disease? Front Immunol. 2019;10:3092. 31993062 10.3389/fimmu.2019.03092 PMC6971046

[6] Ley RE, Turnbaugh PJ, Klein S, Gordon JI. Microbial ecology: human gut microbes associated with obesity. Nature. 2006;444(7122):1022–3. 17183309 10.1038/4441022a

[7] Hammoud AO, Wilde N, Gibson M, Parks A, Carrell DT, Meikle AW. Male obesity and alteration in sperm parameters. Fertil Steril. 2008;90(6):2222–5. 18178190 10.1016/j.fertnstert.2007.10.011

[8] Kahn BE, Brannigan RE. Obesity and male infertility. Curr Opin Urol. 2017;27(5):441–5. 28661897 10.1097/MOU.0000000000000417

[9] Chen H, Luo T, Chen T, Wang G. Seminal bacterial composition in patients with obstructive and non-obstructive azoospermia. Exp Ther Med. 2018;15(3):2884–90. 29456693 10.3892/etm.2018.5778 PMC5795641

[10] Hou D, Zhou X, Zhong X, Settles ML, Herring J, Wang L, et al. Microbiota of the seminal fluid from healthy and infertile men. Fertil Steril. 2013;100(5):1261–9. 23993888 10.1016/j.fertnstert.2013.07.1991 PMC3888793

[11] Mandar R, Punab M, Korrovits P, Turk S, Ausmees K, Lapp E, et al. Seminal microbiome in men with and without prostatitis. Int J Urol. 2017;24(3):211–6. 28147438 10.1111/iju.13286

[12] Jarvi K, Lacroix JM, Jain A, Dumitru I, Heritz D, Mittelman MW. Polymerase chain reaction-based detection of bacteria in semen. Fertil Steril. 1996;66(3):463–7. 8751749

[13] Kermes K, Punab M, Loivukene K, Mandar R. Anaerobic seminal fluid micro-flora in chronic prostatitis/chronic pelvic pain syndrome patients. Anaerobe. 2003;9(3):117–23. 16887698 10.1016/S1075-9964(03)00085-4

[14] Altmae S, Franasiak JM, Mandar R. The seminal microbiome in health and disease. Nat Rev Urol. 2019;16(12):703–21. 31732723 10.1038/s41585-019-0250-y

[15] Monteiro C, Marques PI, Cavadas B, Damiao I, Almeida V, Barros N, et al. Characterization of microbiota in male infertility cases uncovers differences in seminal hyperviscosity and oligoasthenoteratozoospermia possibly correlated with increased prevalence of infectious bacteria. Am J Reprod Immunol. 2018;79(6):e12838. 29500854 10.1111/aji.12838

[16] Weng SL, Chiu CM, Lin FM, Huang WC, Liang C, Yang T, et al. Bacterial communities in semen from men of infertile couples: metagenomic sequencing reveals relationships of seminal microbiota to semen quality. PLoS One. 2014;9(10):e110152. 25340531 10.1371/journal.pone.0110152 PMC4207690

[17] Duranti S, Ferrario C, van Sinderen D, Ventura M, Turroni F. Obesity and microbiota: an example of an intricate relationship. Genes Nutr. 2017;12:18. 28638490 10.1186/s12263-017-0566-2 PMC5473000

[18] Ding N, Zhang X, Zhang XD, Jing J, Liu SS, Mu YP, et al. Impairment of spermatogenesis and sperm motility by the high-fat diet-induced dysbiosis of gut microbes. Gut. 2020;69(9):1608–19. 31900292 10.1136/gutjnl-2019-319127 PMC7456731

[19] Su Y, He L, Hu Z, Li Y, Zhang Y, Fan Z, et al. Obesity causes abrupt changes in the testicular microbiota and sperm motility of zebrafish. Front Immunol. 2021;12:639239. 34248933 10.3389/fimmu.2021.639239 PMC8268156

[20] World Health Organization. WHO laboratory manual for the examination and processing of human semen. 6th ed. Geneva: World Health Organization. 2021.

[21] Terquem A, Dadoune J. Aniline blue staining of human spermatozoon chromatin. Evaluation of nuclear maturation. In:. The sperm cell: fertilizing Power, surface Properties, Motility, nucleus and Acrosome, evolutionary aspects proceedings of the fourth international symposium on spermatology, Seillac, France, 27 June–1 July 1982. Springer; 1983.

[22] Hologlu D, Gunes S, Asci R, Henkel R, Guvenc T. Association among sperm chromatin condensation, sperm DNA fragmentation and 8-OHdG in seminal plasma and semen parameters in infertile men with oligoasthenoteratozoospermia. Andrologia. 2022;54(1):e14268. 34632608 10.1111/and.14268

[23] Kabartan E, Gunes S, Arslan MA, Asci R. Investigating the relationship between *BRCA1* and *BRCA2* genes methylation profile and sperm DNA fragmentation in infertile men. Andrologia. 2019;51(7):e13308. 31095775 10.1111/and.13308

[24] Hekim N, Gunes S, Asci R, Henkel R, Abur U. Semiquantitative promoter methylation of *MLH1* and *MSH2* genes and their impact on sperm DNA fragmentation and chromatin condensation in infertile men. Andrologia. 2021;53(1):e13827. 33112435 10.1111/and.13827

[25] Metin Mahmutoglu A, Gunes S, Asci R, Henkel R, Aydin O. Association of XRCC1 and ERCC2 promoters’ methylation with chromatin condensation and sperm DNA fragmentation in idiopathic oligoasthenoteratozoospermic men. Andrologia. 2021;53(2):e13925. 33355950 10.1111/and.13925

[26] Gupta S, Caraballo M, Agarwal A. Total antioxidant capacity measurement by colorimetric assay. In: Henkel R, Samanta L, Agarwal A, editors. Oxidants, Antioxidants, and Impact of the Oxidative Status in Male Reproduction. London: Academic Press; 2018. p. 57–63.

[27] Klindworth A, Pruesse E, Schweer T, Peplies J, Quast C, Horn M, et al. Evaluation of general 16S ribosomal RNA gene PCR primers for classical and next-generation sequencing-based diversity studies. Nucleic Acids Res. 2013;41(1):e1. 22933715 10.1093/nar/gks808 PMC3592464

[28] Yao Y, Qiu XJ, Wang DS, Luo JK, Tang T, Li YH, et al. Semen microbiota in normal and leukocytospermic males. Asian J Androl. 2022;24(4):398–405. 34916474 10.4103/aja202172 PMC9295480

[29] Garcia-Segura S, Del Rey J, Closa L, Garcia-Martinez I, Hobeich C, Castel AB, et al. Seminal microbiota of idiopathic infertile patients and its relationship with sperm DNA integrity. Front Cell Dev Biol. 2022;10:937157. 35837328 10.3389/fcell.2022.937157 PMC9275566

[30] Bukharin OV, Perunova NB, Ivanova EV, Chaynikova IN, Bekpergenova AV, Bondarenko TA, et al. Semen microbiota and cytokines of healthy and infertile men. Asian J Androl. 2022;24(4):353–8. 34806653 10.4103/aja202169 PMC9295472

[31] Baud D, Pattaroni C, Vulliemoz N, Castella V, Marsland BJ, Stojanov M. Sperm microbiota and its impact on semen parameters. Front Microbiol. 2019;10:234. 30809218 10.3389/fmicb.2019.00234 PMC6379293

[32] Lundy SD, Sangwan N, Parekh NV, Selvam MKP, Gupta S, McCaffrey P, et al. Functional and taxonomic dysbiosis of the gut, urine, and semen microbiomes in male infertility. Eur Urol. 2021;79(6):826–36. 33573862 10.1016/j.eururo.2021.01.014

[33] Aghazarian A, Huf W, Klingler HC, Klatte T. The effect of seminal pathogens on standard semen parameters, sperm kinematics and seminal inflammatory markers. J Reprod Immunol. 2024;161:104183. 38154434 10.1016/j.jri.2023.104183

[34] Cao T, Wang S, Pan Y, Guo F, Wu B, Zhang Y, et al. Characterization of the semen, gut, and urine microbiota in patients with different semen abnormalities. Front Microbiol. 2023;14:1182320. 37293215 10.3389/fmicb.2023.1182320 PMC10244769

[35] Osadchiy V, Belarmino A, Kianian R, Sigalos JT, Ancira JS, Kanie T, et al. Semen microbiota are dramatically altered in men with abnormal sperm parameters. Sci Rep. 2024;14(1):1068. 38212576 10.1038/s41598-024-51686-4 PMC10784508

[36] Wilde J, Allen-Vercoe E. Characterizing prophages in the genus *Fusobacterium*. Anaerobe. 2023;80:102718. 36801248 10.1016/j.anaerobe.2023.102718

[37] Tuominen H, Rautava J, Kero K, Syrjanen S, Collado MC, Rautava S. HPV infection and bacterial microbiota in the semen from healthy men. BMC Infect Dis. 2021;21(1):373. 33882835 10.1186/s12879-021-06029-3 PMC8059035

[38] Ranjan R, Rani A, Metwally A, McGee HS, Perkins DL. Analysis of the microbiome: advantages of whole genome shotgun versus 16s amplicon sequencing. Biochem Biophys Res Commun. 2016;469(4):967–77. 26718401 10.1016/j.bbrc.2015.12.083 PMC4830092

