# Supplementary material for: Evaluation of the semen microbiome for fertility in men with obesity using next-generation sequencing
Source: Basic Clin Androl. 2025 Dec 5;35:47. doi: 10.1186/s12610-025-00294-x (PMC12679728; doi:10.1186/s12610-025-00294-x)
Supplement: Supplementary file 1 — Additional File 1: Supplementary Table 1. Statistical data regarding the demographic information of the patient and control groups. [file 12610_2025_294_MOESM1_ESM.doc]

Supplementary Table 1. Statistical data regarding the demographic information of the patient and control groups

| **Parameter** | **Patients (*n*=13)** | **Controls (*n*=5)** | ***p* value** |
| --- | --- | --- | --- |
| **Age (year)** | | | |
| Mean ± SD | 34.46 ± 7.52 | 38.8 ± 4.27 | 0.1240 |
| Median | 32 | 39 |  |
| **Weight (kg)** | | | |
| Mean ± SD | 103.46 ± 11.28 | 94.2 ± 1.64 | 0.0538 |
| Median | 100 | 95 |  |
| **Height (m)** | | | |
| Mean ± SD | 1.76 ± 0.07 | 1.74 ± 0.06 | 0.8416 |
| Median | 1.78 | 1.72 |  |
| **BMI (kg/m^2^)** | | | |
| Mean ± SD | 33.28 ± 2.04 | 31.26 ± 1.97 | 0.0945 |
| Median | 32.87 | 32.11 |  |

Age, weight, height, and body mass index (BMI) data for the patient and control groups is presented. As intended, no statistically significant differences were observed for these variables, ensuring the comparability of the groups. *Mann Whitney U test (*α*=0,05)
