# Supplementary material for: Evaluation of the semen microbiome for fertility in men with obesity using next-generation sequencing
Source: Basic Clin Androl. 2025 Dec 5;35:47. doi: 10.1186/s12610-025-00294-x (PMC12679728; doi:10.1186/s12610-025-00294-x)
Supplement: Supplementary file 2 — Additional File 2: Supplementary Table 2. Statistical data regarding semen parameters of patient and control groups. [file 12610_2025_294_MOESM2_ESM.doc]

Supplementary Table 1. Statistical data regarding semen parameters of patient and control groups

| **Parameter** | **Patients (*n*=13)** | **Controls (*n*=5)** | ***p* value** |
| --- | --- | --- | --- |
| **Volume (ml)** | | | |
| Mean ± SD | 2.72 ± 0.84 | 3.00 ± 0.71 | 0.2758 |
| Median | 2.80 | 3.00 |  |
| Range | 1.6-5 | 2-4 |  |
| **Sperm Concentration (million/ml)** | | | |
| Mean ± SD | 12.9 ± 16.74 | 30.60 ± 9.34 | 0.0427* |
| Median | 3.00 | 26.00 |  |
| Range | 0-56 | 23-45 |  |
| **Total Sperm Count (million/ejaculate)** | | | |
| Mean ± SD | 36.54 ± 48.96 | 87.60 ± 14.29 | 0.0318* |
| Median | 15.00 | 90.00 |  |
| Range | 0-168 | 69-105 |  |
| **Total Progressive Motile Sperm Count (million)** | | | |
| Mean ± SD | 17.35 ± 27.73 | 43.20 ± 8.25 | 0.0273* |
| Median | 1.50 | 43.00 |  |
| Range | 0-97 | 31-53 |  |
| **Progressive Motile Sperm (A) %** | | | |
| Mean ± SD | 25.00 ± 21.85 | 49.60 ± 4.34 | 0.0568 |
| Median | 20.00 | 51.00 |  |
| Range | 0-58 | 45-54 |  |
| **Non-Progressive Sperm (B)** % | | | |
| Mean ± SD | 16.08 ± 2.3 | 5.00 ± 0 | 1.0000 |
| Median | 5.00 | 5.00 |  |
| Range | 0-5 | 5-5 |  |
| **Immotile Sperm (C) %** | | | |
| Mean ± SD | 53.00 ± 34.94 | 45.40 ± 4.34 | 0.0117* |
| Median | 57.00 | 44.00 |  |
| Range | 0-99 | 41-50 |  |
| **Motility (A+B) %** | | | |
| Mean ± SD | 28.54 ± 23.7 | 54.60 ± 4.34 | 0.0568 |
| Median | 25.00 | 56.00 |  |
| Range | 0-63 | 50-59 |  |
| **Normal Morphology %** | | | |
| Mean ± SD | 3.77 ± 3.85 | 9.60 ± 2.61 | 0.0192* |
| Median | 4.00 | 3.77 |  |
| Range | 0-14 | 6-12 |  |

Semen parameter data for the patient and control groups are presented. Statistically significant differences were observed between the groups in several semen parameters, including sperm concentration, total sperm count, total progressive motile sperm count, percentage of immotile sperm, and normal morphology. *Mann-Whitney U test (*α*=0.05) (Statistically significant expressions are indicated with *)
