# Supplementary material for: Evaluation of the semen microbiome for fertility in men with obesity using next-generation sequencing
Source: Basic Clin Androl. 2025 Dec 5;35:47. doi: 10.1186/s12610-025-00294-x (PMC12679728; doi:10.1186/s12610-025-00294-x)
Supplement: Supplementary file 3 — Additional File 3: Supplementary Table 3. Comparison of chromatin condensation, DFI and TAC between groups. [file 12610_2025_294_MOESM3_ESM.doc]

Supplementary Table 1. Comparison of chromatin condensation, DFI and TAC between groups *T test (*α*=0.05)

| **Histon-Rich Sperm %** | **Patients (*n*=9)** | **Controls (*n*=5)** | ***p* value** |
| --- | --- | --- | --- |
| Mean | 48.15 ± 19.14 | 33.68 ± 15.65 | 0.172 |
| Median | 38.20 | 25.33 |  |
| Minimum | 24.35 | 20.68 |  |
| Maximum | 76.29 | 54.81 |  |
| **DFI** | **Patients (*n*=9)** | **Controls (*n*=5)** | ***p* value** |
| Mean | 13.39 ± 3.27 | 12.10 ± 4.93 | 0.564 |
| Median | 12.62 | 6.80 |  |
| Minimum | 8.43 | 4.13 |  |
| Maximum | 18.56 | 16.24 |  |
| **TAC** | **Patients (*n*=12)** | **Controls (*n*=5)** | ***p* value** |
| Mean | 2.87 ± 0.21 | 2.69 ± 0.20 | 0.127 |
| Median | 2.87 | 2.72 |  |
| Minimum | 2.54 | 2.47 |  |
| Maximum | 3.13 | 2.95 |  |

Sperm chromatin condensation, DNA fragmentation index (DFI), and total antioxidant capacity (TAC) data for the patient and control groups are presented. No statistically significant differences were observed between the groups. *T test (*α*=0.05)
