# Supplementary material for: Evaluation of the semen microbiome for fertility in men with obesity using next-generation sequencing
Source: Basic Clin Androl. 2025 Dec 5;35:47. doi: 10.1186/s12610-025-00294-x (PMC12679728; doi:10.1186/s12610-025-00294-x)
Supplement: Supplementary file 5 — Additional File 5. Detailed results of aniline staining, ELISA and TUNEL analyses. [file 12610_2025_294_MOESM5_ESM.doc]

**ANILIN STAINING RESULTS**

| **Patient Code** | **Anilin-positive sperm count on the first slide** | **Anilin-negative sperm count on the first slide** | **Anilin-positive sperm percentenge on the first slide** | **Anilin-positive sperm count on the second slide** | **Anilin-negative sperm count on the second slide** | **Anilin-positive sperm percentenge on the second slide** | **Total count of anilin-positive sperms** | **Total count of anilin-negative sperms** | **Total percentege of anilin-positive sperms** |
| --- | --- | --- | --- | --- | --- | --- | --- | --- | --- |
| **H3** | 103 | 141 | 42.21 | 80 | 154 | 34.19 | 183 | 295 | 38.28 |
| **H4** | 74 | 157 | 32.03 | 84 | 137 | 38.01 | 158 | 294 | 34.96 |
| **H6** | 163 | 61 | 72.77 | 170 | 43 | 79.81 | 333 | 104 | 76.20 |
| **H7** | 156 | 181 | 46.29 | 138 | 141 | 49.46 | 294 | 322 | 47.73 |
| **H8** | 157 | 54 | 74.41 | 146 | 60 | 70.87 | 303 | 114 | 72.66 |
| **H9** | 134 | 79 | 62.91 | 170 | 65 | 72.34 | 304 | 144 | 67.86 |
| **H11** | 79 | 162 | 32.78 | 105 | 151 | 41.02 | 184 | 313 | 37.02 |
| **H12** | 92 | 191 | 32.51 | 97 | 172 | 36.06 | 189 | 363 | 34.24 |
| **H13** | 76 | 214 | 26.21 | 63 | 217 | 22.50 | 139 | 431 | 24.39 |
| **K1** | 64 | 242 | 20.92 | 63 | 218 | 22.42 | 127 | 460 | 21.64 |
| **K2** | 168 | 118 | 58.74 | 108 | 218 | 33.13 | 276 | 336 | 45.10 |
| **K3** | 173 | 146 | 54.23 | 154 | 124 | 55.40 | 327 | 270 | 54.77 |
| **K4** | 70 | 214 | 24.65 | 71 | 202 | 26.01 | 141 | 416 | 25.31 |
| **K5** | 90 | 294 | 23.44 | 50 | 229 | 17.92 | 140 | 523 | 21.12 |

**ELISA RESULTS**

**Absorbance Values ​​of Samples Diluted at 1:20 Ratio**

|  | **405 nm** | | | | **750 nm** | | | |
| --- | --- | --- | --- | --- | --- | --- | --- | --- |
| **Patient Code** | **1^st^ read** | **2^nd^ read** | **3^rd^ read** | **Average Absorbance** | **1^st^ read** | **2^nd^ read** | **3^rd^ read** | **Average Absorbance** |
| **H2** | 0.4570 | 0.4373 |  | 0.4472 | 0.1673 | 0.1602 |  | 0.1638 |
| **H3** | 0.4673 | 0.3995 |  | 0.4334 | 0.1719 | 0.1430 |  | 0.1575 |
| **H4** | 0.4468 | 0.4578 |  | 0.4523 | 0.1648 | 0.1674 |  | 0.1661 |
| **H5** | 0.4575 | 0.3930 |  | 0.4253 | 0.1705 | 0.1354 |  | 0.1530 |
| **H6** | 0.4148 | 0.4410 | | 0.3955 | | --- | | 0.4171 | 0.1501 | 0.1540 | | 0.1399 | | --- | | 0.1480 |
| **H7** | 0.4895 | 0.4367 |  | 0.4631 | 0.1802 | 0.1620 |  | 0.1711 |
| **H8** | 0.5017 | 0.4611 | | 0.4739 | | --- | | 0.4789 | 0.1872 | 0.1680 | | 0.1791 | | --- | | 0.1781 |
| **H9** | 0.4732 | 0.4594 |  | 0.4663 | 0.1762 | 0.1734 |  | 0.1748 |
| **H10** | 0.4450 | 0.4207 |  | 0.4329 | 0.1587 | 0.1462 |  | 0.1525 |
| **H11** | 0.4442 | 0.3897 |  | 0.4170 | 0.1639 | 0.1318 |  | 0.1479 |
| **H12** | 0.4607 | 0.4732 |  | 0.4670 | 0.1703 | 0.1758 |  | 0.1731 |
| **H13** | 0.4171 | 0.3874 |  | 0.4023 | 0.1423 | 0.1455 |  | 0.1439 |
| **K1** | 0.3950 | 0.4045 | 0.4138 | 0.4044 | 0.1343 | 0.1406 | 0.1486 | 0.1412 |
| **K2** | 0.4691 | 0.4883 | 0.4731 | 0.4768 | 0.1700 | 0.1753 | 0.1768 | 0.1740 |
| **K3** | 0.4240 | 0.4529 | 0.4363 | 0.4377 | 0.1499 | 0.1631 | 0.1584 | 0.1571 |
| **K4** | 0.4528 | 0.4704 | 0.4300 | 0.4511 | 0.1649 | 0.1711 | 0.1569 | 0.1643 |
| **K5** | 0.3773 | 0.4073 | 0.3771 | 0.3872 | 0.1276 | 0.1478 | 0.1321 | 0.1358 |


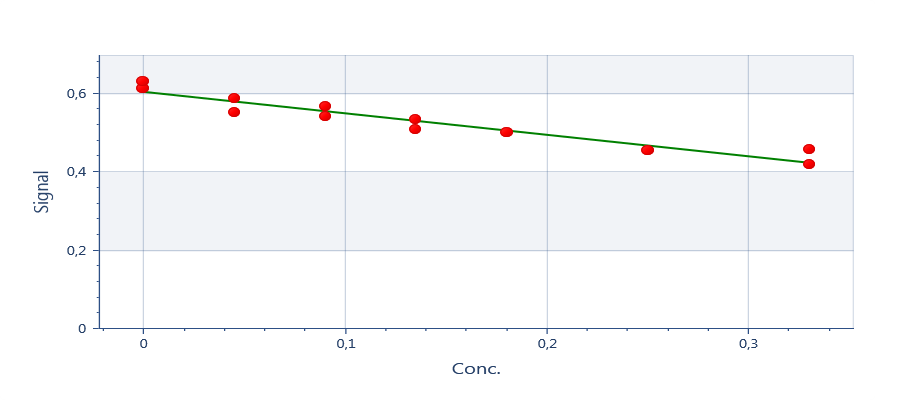
Trolox Standard Curve

**TUNEL** Analysis

| **Patient Code** | **Total DAPI Signal Count** | **Total FITC Signal Count** | **DFI** |
| --- | --- | --- | --- |
| **H3** | 220 | 43 | 16.35 |
| **H4** | 1130 | 104 | 8.43 |
| **H6** | 389 | 46 | 10.57 |
| **H7** | 653 | 93 | 12.47 |
| **H8** | 342 | 54 | 13.64 |
| **H9** | 338 | 68 | 16.75 |
| **H11** | 540 | 78 | 12.62 |
| **H12** | 136 | 31 | 18.56 |
| **H13** | 151 | 19 | 11.18 |
| **K1** | 487 | 77 | 15.81 |
| **K2** | 2834 | 117 | 4.13 |
| **K3** | 234 | 38 | 16.24 |
| **K4** | 218 | 24 | 11.01 |
| **K5** | 630 | 84 | 13.33 |
